# Multi-Site Bundling of Drought Tolerant Maize Varieties and Index Insurance

**DOI:** 10.1111/1477-9552.12344

**Published:** 2019-06-04

**Authors:** Sebastain N. Awondo, Genti Kostandini, Peter Setimela, Olaf Erenstein

**Affiliations:** 1 Sebastain Awondo is an Associate Researcher in the Alabama Center for Insurance Information & Research, Culverhouse College of Business, University of Alabama, Tuscaloosa, AL, USA; 2 Genti Kostandini is an Associate Professor in the Department of Agricultural & Applied Economics, University of Georgia, Griffin, USA; 3 Peter Setimela is with the International Maize and Wheat Improvement Center (CIMMYT), Harare, Zimbabwe, and Olaf Erenstein is with CIMMYT, Texcoco, Mexico

**Keywords:** Africa, drought tolerant maize varieties, mega-environment, multi-site index insurance, multivariate hierarchical Bayes, C11, D80, O33, Q12

## Abstract

Drought Tolerant Maize Varieties (DTMV) and Rainfall Index Insurance (RII) are potential complements, though with limited empirical basis. We employ a multivariate spatial framework to investigate the potential for bundling DTMV with a simulated multi-site and multi-environment RII, designed to insure against mild, moderate and severe drought risk. We use yield data from on-farm trials conducted by the International Maize and Wheat Improvement Center (CIMMYT) and partners over 49 locations in Eastern and Southern Africa spanning 8 countries and 5 mega-environments (dry lowland, dry mid altitude, wet lower mid altitude, low wetland and wet upper mid altitude) in which 19 different improved maize varieties including DTMV were tested at each location. Spatially correlated daily rainfall data are generated from a first-order two-state Markov chain process and used to calibrate the index and predict yields with a hierarchical Bayes multivariate spatial model. Results show high variation in the performance and benefits of different bundles which depend on the maize variety, the risk layer insured, and the type of environment, with high chances of selecting a sub-optimal and unattractive contract. We find that complementing RII with a specific DTMV produces contracts with lower premiums and higher guaranteed returns especially in dry lowland increasing the chances of scaling up RII within this environment.

## 1 Introduction

Rural households in most developing countries experience severe income and food consumption fluctuations due to drought. This vulnerability has the potential to further escalate given projections of more severe and frequent adverse weather conditions due to climate change, and threatens to roll back achievements in global poverty levels (FAO, 2007). The effect of drought is compounded by costly decisions made by farmers who often trade off considerable future gains (from adopting new technology) for reduced drought related losses (Eswaran and Kotwal, 1990; Rosenzweig and Wolpin, 1993; Jacoby and Skoufias, 1997).

Over the last two decades significant resources have been allocated by donors and governments in developing countries to develop drought tolerant maize varieties (DTMV) and weather index insurance (WII) for farmers to manage drought risk.^2^ However, the demand for most WII has been very low with little potential for scalability and sustainability (Barnett *et al.*, 2008; Binswanger-Mikhize, 2012). The low demand has been attributed to credit and cash constraints, competition from informal risk-sharing networks (Hess and Syroka, 2006; Barnett *et al.*, 2008; Molini *et al.*, 2008; Binswanger-Mikhize, 2012), as well as the complex nature of the technology and the uninsured basis risk it creates (Hess and Syroka, 2006; Molini *et al.*, 2008; Clarke, 2011) from poor correlation between yield losses predicted by the index (e.g. based on rainfall recorded at the local meteorological station) and that actually experienced on individual farms.

On the other hand, significantly higher adoption rates of improved (and drought tolerant) maize varieties by farmers are being reported (Cooper *et al.*, 2013; Diiro, 2013; Fisher *et al.*, 2015) whose development requires high upfront cost but a near zero marginal cost of producing DTMV seeds. These seeds provide farmers with some protection against mild to moderate drought. However, as drought severity increases beyond a certain point, the yield advantage of DTMV over less/non-DTMV begins to erode, and at severe drought conditions the yield advantage is zero. On the contrary, WII requires a meager upfront development cost and has the potential to insure households against mild, moderate and severe drought. However, the actuarially fair premiums are costly to farmers and could be perceived as unfair for cash constrained individuals.^3^ Designing WII to cover (more severe) drought risk beyond the point where DTMV is losing its yield advantage is expected to significantly reduce premiums and offer better benefits to farm households as drought severity increases, thus making contracts more attractive. This might spur demand for WII, reduce underinsurance, and improve the pool and mix of the insured making scalability and sustainability more feasible.

In fact, R4 Rural Resilience Initiative (R4) and ACRE Africa, both insurance programmes in Africa with growing demand, credit their success to a holistic approach of risk management which complements WII with quality farm inputs, credits, savings or disaster risk reduction programmes to help vulnerable communities better manage risk.^4^ However, while there are apparent gains in bundling risk management technologies in a systematic approach, research which examines this approach is limited.

Drought tolerant maize varieties have a seemingly complementary relationship with rainfall index insurance (RII). However, this relationship is generally assumed in designing crop insurance contracts without any theoretical or empirical basis. In addition, very little is known about the exact nature of the relationship, including how it varies across space, drought severity levels, and their potential benefits and limitations. To the best of our knowledge, only one study, Lybbert and Carter (2014), has made this connection using a systematic approach. The authors conceptually demonstrated the complementarity between the two technologies using yield data for a single maize variety from a community in Ecuador, a hypothetical drought tolerant maize variety, and a hypothetical rainfall index in a non-spatial framework. Even though they found evidence that supports the complementarity between the two technologies and improved benefits, their findings are limited by the hypothetical assumptions made in their analysis.

Other studies have taken a different approach in investigating interactions between insurance and other risk management technologies. Carriquiry and Osgood (2012) examined the relationship between climate forecasts, insurance and production decisions using a theoretical model of decision-making under risk and uncertainty in the absence of moral hazard, and found that forecast information, even though risk-reducing can induce farmers to use more risk-increasing inputs as well as increase their demand for insurance. Using a randomised control trial involving Indian farmers, Cole *et al.* (2016) also found that in the presence of insurance, educated farmers shift production toward higher returns using higher-risk cash crops. Exploiting the interaction between members of a group and the covariate risk they face, Traerup (2011), Dercon *et al.* (2014) and de Janvry *et al.* (2014) conceptually argued that the demand for WII can be improved by selling insurance to informal risk-sharing groups instead of individual farmers. Skees and Barnett (2006) and Skees *et al.* (2007) investigated bundling WII with micro loans to facilitate farmers’ access to credit with positive results. However, access to credit does not necessarily ensure acquisition of a drought tolerant variety that highly complements the risks insured by a specific WII, and except Lybbert and Carter (2014), no other study has considered the potential benefits and limitations offered by complementing DTMV and WII jointly in a systematic approach.

In this study, we investigate the potential for bundling DTMV with a simulated multi-site and multi-environment rainfall index insurance (RII) to better insure households against drought risk and to facilitate scaling up and sustainability of farm risk management programmes. Specifically, using daily rainfall and yield data from unique on-farm trials conducted by the International Maize and Wheat Improvement Center (CIMMYT) and partners over 49 locations in Eastern and Southern Africa spanning 8 countries (Ethiopia, Kenya, Malawi, Mozambique, Tanzania, Uganda, Zambia and Zimbabwe) and 5 maize mega-environments (dry lowland, dry mid altitude, wet lower mid altitude, low wetland and wet upper mid altitude), we extend the framework proposed by Lybbert and Carter (2014) to a multivariate spatial framework and investigate the potential relationships and benefits from complementing 19 improved maize varieties with a simulated multi-site and multi-environment RII, designed to insure against mild to severe drought risk layers (quantiles). Thus, bundling in this study entails choosing the optimal combination of a drought tolerant maize variety and RII to offer in each mega-environment given its drought risk quantiles (that is, the index point beyond which the contract begins to pay out).

The remainder of the paper is structured as follows. In the next section, we discuss the motivation behind our model framework. In section 3, we summarise the multivariate spatial hierarchical Bayes model used in predicting yields over different drought severity conditions. This section also summarises the rainfall simulator. We discuss and summarise data use in section 4, and present and discuss the results in section 5. Finally, we conclude with major findings, policy implications and potential for future research.

## 2 Motivation for Model Framework

Maize is a major food crop in most countries in Eastern, Southern and West Africa and accounts for about 53% of all cereals (FAO *et al.*, 2012) and 70% of total caloric intake (Langyintuo *et al.*, 2010). Maize production in these regions is mostly rainfed and highly vulnerable to adverse weather conditions. Yield losses associated with drought range between 10% and 50% on average. Losses are remarkably higher in environments with low soil fertility and other biotic stresses (FAO *et al.*, 2012). Under likely climate change scenarios, more frequent adverse weather conditions have been predicted making these regions increasingly vulnerable to food insecurity and poverty.

From an agronomic standpoint, DTMV have the ability to significantly reduce yield losses under specific water stress levels, but their potential varies across agro-ecological regions. Breeding drought tolerant maize traits adapted to specific regions requires extensive on-farm trials posing both financial and systemic constraints. The specific stress of interest in the trial (drought) may not be observed at the location during the season in which the trial was conducted. When this occurs, genotype performance and stability across environments cannot be properly evaluated using data from such trials. While incorporating conventional maize breeding with managed stress screening has been shown to be viable, on-farm trials remain essential in evaluating the performance and stability of genotypes in actual environments. In addition, data from such trials in which several DTMV are consistently tested over space and time are rarely available, posing estimation problems.

The data used in this study are taken from on-farm trials that were conducted in 2011 and involved 19 different improved maize varieties including hybrids, and open pollinated maize varieties. During the trials, 13 locations actually experienced water stress levels, creating data limitations. In addition, there is a general absence of long time series for weather data in most developing countries including the regions in this study, which adds to the data constraints. Commercial property and casualty insurers require at least 30 years of time series data to properly quantify the probability of dry spell and loss.

We address the data limitations by developing a model that allows us to effectively predict yields across space and time.^5^ Specifically, we use a hierarchical Bayes multivariate spatial model that also allows for spatial correlation and cross correlation among trial sites and agro-mega environments. We simulate correlated space-time growing seasonal rainfall over the 49 locations using a multi-site rainfall simulator (Wilks, 1998), and use it in the posterior predictive distribution to generate yields for the 19 maize varieties under different severity levels of water stress. In the next step, following Lybbert and Carter (2014), we calibrate RII with five different trigger points (reflecting mild to severe drought levels at which DTMV is a potential complement) that correspond to the 50%, 45%, 35%, 25% and 10% rainfall index and yield quantiles by agro-mega environment.

To investigate the potential complementarity of DTMV with RII, we examine stability of yields, yield differentials, fair premium rates, certainty equivalent and changes in certainty equivalent revenue afforded by each maize variety across trial locations, agro-mega environments at all five drought severity quantiles corresponding to rainfall trigger levels for the index insurance. In the final part of our analysis, we estimate and compare the correlation between the indices and the yield and yield differential by location and agro-mega environment.

In practice, bundling DTMV and RII requires the index to be strongly correlated with yield loss, and proper knowledge of the net yield profile for the drought tolerant variety compared to a competing alternative variety. Maize varieties have different tolerance to drought and potentially high variation in yield losses from one variety to another. Moreover, these losses likely vary from one agro-climatic environment to another, making it diffcult to find a good index over a large area. This serves as a limitation to scale up and sustain index insurance programmes. However, a key positive feature is that, a multi-site index can take advantage of negative correlation in losses between sites caused by weather patterns to create an index with lower premiums (IRI, 2009). Such patterns have been reported between Eastern and Southern African regions. In order to accurately develop a multi-site index of this nature one needs to correctly account for spatial and temporal variation in rainfall and yields of specific DTMV.

## 3 Model Framework

For completeness, we present a summary of the multivariate spatial hierarchical Bayes model employed to simulate maize yields, and refer to Finley *et al.* (2007, pp. 3–8) for detailed derivation of the model. For simplicity, we first introduce a bivariate yield spatial model involving two locations before generalising to an *m*-variate model over *n* locations. A simple bivariate spatial model involving two maize varieties and a single predictor over two locations follows:

(1) $$\begin{array}{l} \left[\begin{array}{c} y_{11}\\ y_{12}\\ y_{21}\\ y_{22} \end{array}\right]=\left[\begin{array}{cccc} 1 & x_{1} & 0 & \\ 1 & 0 & x_{1} & \\ 1 & {\begin{array}{c} x \end{array}}_{2} & 0 & \\ 1 & 0 & x_{2} & \end{array}\right]\left[\begin{array}{c} \beta_{0}\\ \beta_{1}\\ \beta_{2} \end{array}\right]+\left[\begin{array}{c} w_{11}\\ w_{12}\\ w_{21}\\ w_{22} \end{array}\right]+\left[\begin{array}{c} \epsilon_{11}\\ \epsilon_{12}\\ \epsilon_{21}\\ \epsilon_{22} \end{array}\right],\\ 𝕐=𝕏\beta +𝕎+\epsilon \end{array}$$

where 𝕐 is a 4 × 1 vector of yields; *y*_11_ is the yield of variety 1 in location 1, *y*_12_ is the yield of variety 2 in location 1, and so on; 𝕏 is a 4 ×3 matrix with *x*_1_ and *x*_2_ as cumulative rainfall during maize growing season in locations 1 and 2, respectively; ***β*** is 3 ×1 vector of parameters with *β*_1_ and *β*_2_ representing the effect of rainfall on variety 1 and 2, respectively; *w*_11_ and *w*_21_ are spatial covariance of maize variety 1 while *w*_12_ and *w*_22_ are spatial covariance of variety 2. 𝕎 is a 4 ×1 vector of the spatial covariance of yields (𝕐) between site 1 (*i*) and site 2 (*j*) assumed to come from a multivariate normal distribution with mean zero (4 ×1 vector) and covariance (4 ×4 vector) 𝕂(*i*; *j*; θ); that is 𝕎 ~ *MVN*(0, 𝕂(*i, j*; θ)), θ is the parameter in the spatial correlation function that controls the correlation decay process between two locations as distance between the location changes. Similarly, ε ~ *MVN*(**0**; *I*_2_ ⊗ ψ) with a 4 ×1 vector of mean zero, *I*_2_ is a 2 × 2 identity matrix, Ψ a 2 × 2 diagonal matrix with $\left[\tau_{1}^{2},\tau_{2}^{2}\right]$ on the diagonal, $\tau_{1}^{2}$ and $\tau_{2}^{2}$ is the variance of variety 1 and 2, respectively.^6^

For *m* maize varieties over *n* sites, 𝕐 is an *mn* × 1 vector and 𝕏 ^𝕋^ is a *mn* × *q* matrix such that $q=1+\Sigma_{i=1}^{m}p_{i}$, where *p_i_* is the number of predictors for maize variety *i*; an *mn* × 1 vector 𝕎 ~ *MVN*(0, Σ_***W***(***θ***)_) with an *mn* × 1 vector of means zero and an *mn* × *mn* cross-covariance matrix $\Sigma_{w\left(\theta \right)}{=[𝕂\left(i,j;\theta \right)]}_{i,j=1}^{n}$ where 𝕂(*i, j*; θ) represents the (*i, j*)th block with *m* × *m* dimension. The error, ε ~ *MVN*(**0**; *I*_*n*_ ⊗Ψ) where *I_n_* is a *n* × *n* identity matrix, ⊗ is the Kronecker product, Ψ an *m* × *m* diagonal matrix with $\left[\tau_{1}^{2},\tau_{2}^{2},\ldots ,\tau_{m}^{2}\right]$ on the diagonal. Thus, implying the covariance, *I_n_* ⊗Ψ, is an *mn* × *mn* block diagonal matrix. Therefore, the covariance matrix of the observed yields, 𝕐 = **Σ**_***W***(***θ***)_ + 𝕀_*n*_ ⊗ Ψ.

Estimation is only feasible when **Σ**_***W***(***θ***)_ is positive and semi-definite. Following Wackernagel (2003), we obtain feasible choices of **Σ**_***W***(***θ***)_ using coregionalisation whereby is linearly transformed using an *m* x*m* non-singular spatial varying matrix (**A**), thus allowing more flexible forms of spatial correlation structures (e.g. Mat"ern, spherical, exponential, Gaussian) to be estimated. Following Finley *et al.* (2007), **A** is identified as the Cholesky square root of 𝕂 = (*i, j*; θ) which we take as its lower triangular matrix. For *m* maize varieties across *n* sites, the transformation of the cross-covariance matrix is $\Sigma_{\overset{˜}{W}}=𝕁E_{w}{𝕁}^{T}$ where $𝕁=I_{\text{n}}⊗\text{A}$ is an *mn* × *mn* matrix.

### 3.1 Hierarchical Bayes model

Following Gelman *et al.* (2003) and Finley *et al.* (2007, 2008), we employ a hierarchical Bayes approach with a generalised specification that allows the fitting of different classes of spatial dependence processes using Gibbs sampler with Metropolis updates. To induce such flexibility while ensuring that **Σ**_***W***(***θ***)_ is symmetric and positive definite, we use its linear transformation, $\Sigma_{\overset{˜}{W}}$, where $A={𝕂}^{\frac{1}{2}}\left(i,j;\theta \right)$ is the Cholesky square-root of 𝕂(*i, j*; **θ**)taken as its lower triangular-matrix. The generic model is specified as:

(2) $$\begin{array}{cc} 𝕐=𝕏\beta +\text{𝕁𝕎}+\epsilon , & \;\epsilon \sim MVN\left(0,I_{\text{n}}⊗\Psi \right) \end{array}$$

Equation (2) implies that 𝕐 ~ *MVN* (𝕏**β**; 𝕁Σ_𝕎_𝕁^T^ + *I*_*n*_⊗ Ψ). We assume β ~ *MVN* (μ_β_,Σ_β_), and the errors across locations are independent and follow an inverse gamma distribution, that is $\tau_{i}^{2}\sim IG\left(a_{i},b_{i}\right)$ for all $\tau_{i}^{2}$ in the diagonal of Ψ. The posterior distribution is then given as:

(3) P(Θ|Data)∝P(β)P(𝕁)P(θ)P(Ψ)P(𝕐|β,𝕁,θ,Ψ),

where **Θ** = (***β***, 𝕁, θ,Ψ). To simulate the joint posterior, the β parameters are given a diffuse prior, while the dispersion parameters ($\tau_{i}^{2}$) takes on inverse gamma diffuse priors. The structure of 𝕁 depends on **A** since 𝕁 = *I*_*n*_ ⊗ *A*, and we assign an inverse-Wishart prior on **AA**^T^. An informative prior on the correlation decay parameter (θ)is set so that the prior means imply a spatial range of ¼ of the maximum distance between two locations in the sample (Finley *et al.*, 2008). Smoothness parameters (μ) for the Mat^0^ ern model are assumed to be uniformly distributed between 0 and 2. The Markov Chain Monte Carlo (MCMC) algorithm draws and updates β from its full conditional using Gibbs sampling, while 𝕁, θ, Ψ are updated using Metropolis-Hastings steps. We simulated *L* (10,000) samples of the joint posterior distribution and discarded the first 7,500. Then, the posterior distribution of 𝕎 is recovered from the following predictive distribution:

(4) P(𝕎|Data)∝∫P(𝕎|Θ,Data)P(Θ|Data)dΘ.

First, we draw L samples of **Θ** from *P*(**Θ**|*Data*), and then use them in *P*(𝕎|**Θ**; *Data*) to draw L samples of the spatial random field (𝕎). To predict yields in n^*^ locations using the 500 simulated rainfall samples over the growing season, we use the predictive distribution:

(5) P(𝕐*|Data)∝∫P(𝕐*|Θ,Data)P(Θ|Data)dΘ.

To identify the best model, we estimate 28 separate models made up of different combinations of predictors and spatial correlation functions (exponential, Gaussian spherical and Mat′ern), and compare their deviance information criterion (DIC), effective number of parameters (pD), sum of squared error (G), deviance criterion (D) (Gneiting and Raftery, 2007) and a predictive loss function (Gelfand and Ghosh, 1998). The latter criterion (D = G + P) minimises the loss function using both a goodness-of-fit term (G = sum of squared error) and a penalty criterion (P) for adding more predictors in the model. The results for the selection process are presented in Table A1 in the online Appendix. Based on these criteria, we selected the hierarchical Bayes model with a Mat′ern spatial correlation structure and **X_6_** as the matrix of predictors. The matrix of predictors (**X_6_**) includes cumulative rainfall (PRCP) over the growing season and four indicator (dummy) variables for agro-mega environments taking dry lowland as the base group. Therefore, 𝕂(*i*; *j*; **θ**) is now:

(6) $$\rho \left(i,j;\theta ,\mu \right)=\frac{1}{2^{\mu -1}\Gamma \left(\mu \right)}{\left(\left\|s_{i}-s_{j}\right\|\theta \right)}^{\mu }\hbar_{\mu }\left(\left\|s_{i}-s_{j}\right\|\theta \right);\theta >0,\mu >0$$

where θ controls the decay in spatial correlation and *μ* is a smoothness parameter with higher values yielding smoother process realisations; Γ is the gamma function and *ħ*_*μ*_ is a Bessel function of order *μ*; and ||s_**i**_ − s_**j**_|| is the Euclidean distance between site *i* and *j*.^7^

We cross validate the final model by randomly selecting and excluding data from 6 (of 49) locations and estimate the model with 43 observations. Predictions were then made for the excluded locations and the results compared with the actual yields. Plots of predicted yields versus actual yields, shown in Figure S1 in the online Appendix, overall show good prediction of the observed yields indicating strong potential for predicting out of sample, and thus in evaluating the performance of DTMV under water stress conditions that have not been observed.

We recognise that an alternative model with different effects on the same varieties across different locations would be preferable. However, estimating a spatial model with this specification proves to be much more cumbersome with little hope of converging. The rejection sampling stage within the Metropolis Hastings algorithm is very ineffcient, with improvements in the parameters made at an extremely low rate (<0.005%). We successfully simulated 10,000 draws, which took over 25 times longer than our current specification. A summary of the parameters revealed little spatial correlation; the 95% credible intervals for most of the spatial correlation parameters indicate they are not different from zero. Additionally, the majority of the 980 additional parameters, to capture differing effects, were not statistically different from zero.

### 3.2 Multi-site rainfall simulator

Synthetic daily rainfall samples for 500 years were generated over the 49 locations using a first-order two-state Markov chain (Wilks, 1998). The approach uses serially independent and spatially correlated random numbers, which are then employed individually to generate daily precipitation occurrences and amounts equivalent to time series at each site. Satellite daily rainfall data from 1983 to 2013 at a 10 km by 10 km spatial resolution from the National Oceanic and Atmospheric Administration (NOAA) are used in the simulation. A precipitation threshold of 0.01 inches is used to calibrate the observed daily rainfall occurrences into dry or wet state, and the Markov transition probabilities are derived from direct frequency estimates of the four states (dry-wet, dry-dry, wet-wet and wet-dry). To model the rainfall amounts, we use a two-parameter gamma distribution instead of a mixed exponential distribution as in Wilks (1998). The former has been found to fit rainfall data in Africa and Middle Eastern regions better (Mhanna and Bauwens, 2012).^8^ Next, we use the synthetic rainfall generated to calibrate the rainfall index in each mega-environment. We calibrate the rainfall indices for five (10%, 25%, 35%, 45% and 50%) drought risk quantiles based on a single-phase maize growing season.^9^ The resulting index (*I_sh_*) for drought risk quantile (trigger) *h* in mega-environment *s* is given by:

(7) $$I_{sh}=\left\{1-\frac{R_{s}}{R_{sh}}\right\}$$

where $R_{st}=\sum_{t=Pd}^{Hd}r_{st}$ is the cumulative rainfall amount over the maize growing season in time *t*, that is from the planting date (*Pd*) to harvesting date (*Hd*) predicted at environment *s* for the *t*th time period, *R_sh_* is the *h*th quantile of cumulative rainfall that serves as a trigger in environment *s* below which indemnities are paid.^10^ By using observed planting dates for each farm field, the index directly allows for shifting ‘sowing windows’ across space making the contracts more reliable. Each quantile determines a layer of drought risk covered, thus allowing for five separate potential risk layers that can complement the drought tolerant traits in a given maize variety. Different RII policies are obtained by varying the guaranteed quantile level. The higher the trigger (quantile) the more risk layers are covered including the catastrophic layer, while lower trigger levels will leave out mild to moderate risk layers which occur more frequently. Therefore, complementing DTMV with RII is equivalent to finding the rainfall trigger (quantile) such that the excluded upper risk layer(s) are covered by the drought tolerant traits in a maize variety while the lower ones are insured with the RII. However, to evaluate the performance of the bundle for an index insurance based on a specific drought tolerant maize variety requires comparing the outcome of a similar index when applied to a non/less drought tolerant maize variety (baseline).

In this study, we take a general approach to investigate the potential complementarity between DTMV and the rainfall index. Specifically, we explore five rainfall quantile (triggers) levels selected to disaggregate drought risk in mega-environments into mild, moderate, and severe risk layers allowing a non-drought tolerant, moderately drought tolerant, and a highly drought tolerant maize to complement a RII design to insure mild, moderate, and severe drought risk, respectively.^11^ The five cumulative rainfall quantiles (triggers) are 50%, 45%, 35%, 25% and 10%. The index insurance based on the 50% and 45% quantiles seeks to insure against mild drought, that based on the 35% quantile insures against moderate drought, and the 25% and 10% quantile seeks to insure against severe drought risk. We expect DTMV to perform relatively well compared to the baseline variety under mild to moderate drought conditions, thus complementing an index insurance with a lower rainfall trigger to insure households against all drought risk layers at an affordable rate. The yield loss for maize variety $i\left({\overset{˜}{L}}_{ish}\right)$ based on the *h*th quantile in a mega-environment *s* is estimated using:

(8) $${\overset{˜}{L}}_{ish}=Max\left({\overset{˜}{I}}_{sh}\times {\overset{˜}{Y}}_{ish},0\right).$$

where ${\overset{˜}{Y}}_{ish}$ is the *h*th yield quantile of maize variety *i* in environment *s*. The corresponding actuarially fair premium rate is derived as^12^:

(9) $${\overset{˜}{P}}_{ish}={\overset{˜}{L}}_{ish}/{\overset{˜}{Y}}_{ish}.$$

The revenue (*R_ish_*) obtained from growing variety *i* in environment *s* covered with insurance that triggers at the *h*th quantile is derived as:

(10) $$R_{ish}=\overset{˘}{P}\left(y_{is}-{\overset{˜}{P}}_{ish}+{\overset{˜}{L}}_{ish}\right),$$

where *y_is_* is the predicted yield and $\overset{˘}{P}$ is market price or cost per unit of production. To facilitate the comparison of results across countries (with different currencies), we set $\overset{˘}{P}$ to 1. To estimate certainty equivalent (*CE_ish_*) revenue of maize variety *i* in environment *s* under a contract that triggers at the *h*th quantile, we used a constant relative risk aversion utility function $U\left(R_{ish}\right)=R_{ish}^{1-\lambda }/\left(1-\lambda \right)$ with a risk aversion parameter (λ) of 2 based on estimates found in the literature:

(11) $$\begin{array}{ccc} {CE}_{ish} & = & U^{-1}\left(\int U\left(R_{ish}\right)f\left(y_{is}\right)d\left(y_{is}\right)\right)\\ & = & {\begin{array}{c} \left[\left(1-\lambda \right)\left(\frac{1}{k}\sum_{ish}^{k}U\left(R_{ish}^{\left(f\right)}\right)\right)\right] \end{array}}^{\frac{1}{\left(1-\lambda \right)}}, \end{array}$$

where *k* (=500), is the number of draws. The yield differential ($\Delta {\overset{˜}{Y}}_{ish}$) in each environment *s* based on an insurance with trigger *h* is derived as the difference in predicted yield between variety *i* and a baseline variety *j*; $\Delta {\overset{˜}{Y}}_{ish}={\overset{˜}{Y}}_{ish}-{\overset{˜}{Y}}_{ish}$. In an attempt to capture the possible range of variation in the yield differential and benefits of each bundle between varieties with different degrees of tolerance to drought in each environment, we consider all 18 remaining varieties as a potential baseline variety for any given variety *i*. Finally, we estimate and compare correlations between the indices, yield, and yield differentials. For illustrative purposes, we present results involving selected maize varieties based on a single baseline maize variety across all environments.

From a practical standpoint, these analyses are comparable to using all the rainfall data collected from a network of meteorological stations within the mega-environments to calibrate a single index for the environment. Bundling entails choosing the optimal combination of drought tolerant maize variety and RII to offer in each mega-environment given a drought risk quantile.

## 4 Data

Data for this study come from on-farm trials conducted by CIMMYT and partners in 2011 in 49 farmers’ fields under famer management across 8 countries in Eastern and Southeastern African countries. The 49 farm fields were associated with five different agro-mega environments: dry lowland, dry-mid altitude, wet-lower-mid altitude, low wetland and wet-upper-mid altitude. Nineteen different improved maize varieties including hybrids, open-pollinated varieties, commercial varieties and local varieties (simply referred to here as DT1 to DT19) plus one local maize variety were tested on a farm plot at each location. Each farm represented a block in a randomised complete-block design.

During the year that the trials were conducted only 13 locations actually experienced water stress levels in 2011, making it diffcult to properly evaluate genotype performance and stability under various drought severity levels. In addition, the local variety, which varies by location, makes it diffcult to model in a multivariate framework with the other 19 varieties. The results presented below are based on 19 varieties excluding the local variety.

Satellite daily rainfall data from 1983 to 2013, used to simulate rainfall samples for 500 years, over the locations and mega-environments are obtained from the National Oceanic and Atmospheric Administration (NOAA). The rainfall estimates are obtained from a high gridded spatial resolution (10 km × 10 km) by blending gauge and satellite information. Using the planting and harvesting dates and geographical coordinates for each location, we calculate cumulative rainfall (PRCP) over the growing season. Summary results for the data and simulated rainfall across the 49 locations are presented in Tables 1 and 2, respectively.

**TABLE 1:** Summary of yields (t/ha) and rainfall

Maize variety	Mean (SE)	Min	Max	Variable	Mean (SE)	Min	Max
DTI	3.2 (1.8)	0.47	8.96	DT14	3.6 (2.0)	0.19	11.07
DT2	3.1 (1.8)	0.27	8.29	DT15	3.6 (1.9)	0.25	7.90
DT3	3.6 (2.0)	0.58	11.46	DT16	3.5 (2.1)	0.00	8.68
DT4	3.4 (1.9)	0.26	9.51	DT17	3.7 (2.2)	0.29	12.13
DT5	3.6 (2.2)	0.37	11.56	DT18	3.4 (2.0)	0.54	10.35
DT6	3.3 (1.8)	0.20	9.16	DT19	3.8 (2.4)	0.47	11.21
DT7	3.9 (2.6)	0.47	14.38			
DT8	3.8 (2.3)	0.68	10.88	PRCP	590.3 (178.5)	176.43	1,173.9
DT9	4.0 (2.4)	0.42	12.52	Dry lowland	0.08	0	1
DT10	4.2 (2.3)	0.62	11.90	Dry-mid alt.	0.08	0	1
DT11	4.6 (2.6)	0.51	12.36	Wet-lower-mid-alt.	0.41	0	1
DT12	3.7 (1.8)	0.16	8.96	Wet lowland	0.41	0	^1^
DT13	4.0 (2.5)	0.31	11.95	Wet-Upper-mid alt.	0.39	0	1

**TABLE 2:** Summary of simulated cumulative rainfall over maize growing season by location

Location	*PRCP* (SE)	Min	Max	Location	*PRCP* (SE)	Min	Max
Bikita	389.6 (84.4)	193.5	764.1	ChipokaEPASal.	533.1 (90.0)	296.5	915.4
Bikita	354.7 (92.2)	129.8	680.6	PhalulaBalaka	487.4 (84.2)	267.3	726.0
Bikita	359.6 (89.6)	146.0	662.1	ChipokaEPASal2.	573.8 (94.6)	338.6	970.1
Bikita	350.8 (96.9)	94.2	668.3	Chisamba	551.6 (99.1)	299.8	866.8
Bikita	330.6 (89.0)	107.4	621.2	LusakaWest	599.2 (104.5)	303.5	987.2
Bikita	351.2 90.6)	116.3	640.3	MonzeEast	592.2 (103.5)	314.6	928.9
Zaka	369.0 (80.2)	170.0	647.1	Monze	506.1 (101.0)	217.5	782.1
Zaka	317.4 (86.0)	95.3	648.4	Matsinnho-Gond.	604.7 (137.0)	315.6	981.6
Gokwe	584.1 (98.1)	277.6	856.4	VanduziManica	546.7 (122.2)	220.0	1,011.6
Gokwe	584.1 (100.2)	288.0	836.0	Gondola-Caf.	529.8 (127.8)	170.4	919.0
Gokwe	603.6 (103.2)	347.0	882.9	Iganga	571.0 (77.4)	368.0	795.8
Gokwe	571.0 (105.8)	301.0	889.8	Gulu	685.6 (97.6)	421.9	966.1
Mtoko	572.0 (110.4)	260.0	954.1	Masindi	579.3 (88.5)	336.9	816.0
Mtoko	574.5 (110.2)	313.9	898.4	Kipini	225.3 (83.6)	40.4	601.8
Mtoko	586.3 (115.2)	330.5	1,006.6	Wakiso	464.5 (70.7)	283.7	670.0
Mtoko	587.6 (107.2)	320.6	899.1	Malava	824.7 (99.1)	530.2	1,306.5
Mrewa	665.6 (128.1)	330.4	1,073.1	Bungoma	705.9 (87.2)	487.5	990.3
Mrewa	616.4 (115.2)	282.6	951.8	Alupe	668.7 (82.5)	451.5	913.8
Mrewa	628.1 (123.3)	354.6	1,020.0	KibosPrison	505.5 (70.5)	307.2	715.7
Mrewa	612.89 (116.0)	273.6	992.5	Ethiopia1	268.3 (64.7)	124.6	482.9
UlongaEPA	623.1 (96.1)	387.7	963.0	Ethiopia2	295.4 (71.3)	99.8	527.9
RiviriviEPA	622.3 (110.6)	283.6	974.6	Bofa	266.8 (65.0)	130.7	513.4
Golomoti	589.2 (98.9)	316.7	948.3	Bomangombe	109.3 (39.0)	19.8	262.6
GolomotiEPA	632.9 (99.9)	309.1	977.8	Ethiopia3	324.6 (78.6)	84.2	612.5
Chipoka	587.2 (94.5)	351.6	999.4				

Maize yields range from 0 t/ha for DT16 to 14.38 t/ha for DT7 while mean yields range from 3.12 t/ha for DT2 to 4.57 t/ha in DT11 with relatively high variation ranging from 1.78 t/ha to 2.62 t/ha. Cumulative precipitation over the growing season over all sites range from 176 mm to 1,174 mm with a mean of 590 mm. On the other hand, the 500 simulated rainfall samples at each location range from 20 mm in Bomangombe to 1,307 mm in Malava with expected value ranging from 109 mm in Bomangombe to 825 mm in Malava. Similarly, standard deviations for the expected rainfall range from 40 mm in Bomangombe to 137 mm in Matsinnho-Gond.

## 5 Results and Discussions

Summary of the posterior distribution of model parameters is presented in Table A2 in the online Appendix. The 95% credible interval for cumulative rainfall (*β_PRCP_*) (and several mega-environments) indicates a high probability that the parameters are different from zero. Similarly, credible intervals for μ, θ and Ψ show high probability of the estimates being greater than zero. In the case of θand μ,it shows high spatial correlation in yields across the locations. These results indicate that our restricted spatial model captures most of the systemic variation in maize varieties across different locations. Credible interval estimates for θshow four distinct spatial trends for the DTMV ranging between 4.12 and 12. Those with wide range tend to have the least smooth spatial distribution based on estimates of μ. Estimates of non-spatial variance (Ψ) for each variety range from 0.24 to 2.28. Overall, cumulative rainfall, mega-environment type and spatial correlation are good predictors for DTMV yields.

### 5.1 Stability of DTMV yields, net yields and Index performance

For illustrative purposes, we present results involving four representative maize varieties (DT3, DT6, DT11 and DT17) with DT11 representing the baseline maize variety in each environment. We also focus our analysis on median and lower quantiles since insurance is typically designed for these risk layers.

A summary of the simulated yields is reported in Table A3 in the online Appendix while Table 3 summarizes the yield differential between each of the three varieties (DT3, DT6, DT17) and DT11 (our base).

**TABLE 3:** Summary of yield differential between DT11 and DT3, DT6, DT17

Environment	Variety	2.50%	10%	25%	35%	45%	50%
Dry lowland	DT3	0	0.17	0.93	0.84	0.7	0.62
Dry lowland	DT6	0.87	1.93	2.49	2.25	1.91	1.71
Dry lowland	DT17	0	1.15	2.01	2	1.98	1.97
Dry-mid alt.	DT3	0	0.38	0.15	−0.32	−0.82	−1.07
Dry-mid alt.	DT6	0.66	1.49	1.16	0.7	0.31	0.14
Dry-mid alt.	DT17	0	0.16	−0.04	−0.5	−0.98	−1.22
Wet-lower-mid alt.	DT3	0.29	0.49	0.28	0.12	−0.07	−0.19
Wet-lower-mid alt.	DT6	0.04	0.19	0.64	0.64	0.43	0.29
Wet-lower-mid alt.	DT17	0	−0.43	−0.04	−0.15	−0.32	−0.4
Low wetland	DT3	0.72	1.66	1.36	1.13	0.79	0.61
Low wetland	DT6	1.65	2.42	2.03	1.78	1.47	1.32
Low wetland	DT17	0	0.26	0	−0.22	−0.54	−0.7
Wet-upper-mid alt.	DT3	0.14	0.54	0.74	0.82	0.88	0.88
Wet-upper-mid alt.	DT6	0.15	0.37	0.05	−0.07	−0.1	−0.11
Wet-upper-mid alt.	DT17	0	0.3	0.48	0.41	0.37	0.36

Results in Table A3 show substantial variation in yield performance amongst the maize varieties both within and across mega-environments, thus presenting a need for market insurance. Overall, the reported results across all mega-environments indicate that DT11 (our base) is the least drought tolerant maize variety while DT6 is the most drought tolerant variety.

Results reported in Table 3 show that the yield differential between DT6 and DT11 in dry lowland and wet-lower mid-altitude increases from the 50% up to the 25% quantile, and decreases thereafter. In the dry mid-altitude and low wetland, the yield differential increases up to the 10% quantile before decreasing. Overall, the yield differential tapers to zero after the 10% quantile, indicating that DT6 loses its potential drought tolerant yield advantage over DT11 beyond this point. These results suggest that insuring the 50% up to the 25% or 10% yield quantile of DT6 in dry environments with a RII would complement the drought tolerant traits in DT6 to produce optimal benefits. This is feasible if there is a strong correlation between the rainfall index and the yield (differential) in each environment.

Table A4 in the online Appendix reports the correlation between the rainfall index and maize yields while Table 4 reports the correlation between the index and yield differential. Results in both tables show high variation in the degree of correlations amongst the varieties both within and across mega-environment. The strongest and statistically significant correlation (at the 5% level) is recorded in dry lowland and dry mid-altitudes while the least is recorded in wet lowland and wet-lower mid-altitude, suggesting that dry lowland and dry mid-altitudes environments offer the greatest potentials for bundling DTMVs and RII.

**TABLE 4:** Correlation between yield differential and rainfall index (*P*-value in parentheses)

Environment	Variety	10%	25%	35%	45%	50%
Dry lowland	DT3	0.0	0.31	0.30	0.29	0.29
		(0.00)	(0.00)	(0.00)	(0.00)	(0.00)
Dry lowland	DT6	0.48	0.46	0.45	0.44	0.43
		(0.00)	(0.00)	(0.00)	(0.00)	(0.00)
Dry lowland	DT17	0.19	0.19	0.20	0.20	0.20
		(0.00)	(0.00)	(0.00)	(0.00)	(0.00)
Dry-mid alt.	DT3	0.38	0.38	0.38	0.37	0.37
		(0.00)	(0.00)	(0.00)	(0.00)	(0.00)
Dry-mid alt.	DT6	0.69	0.68	0.68	0.68	0.68
		(0.00)	(0.00)	(0.00)	(0.00)	(0.00)
Dry-mid alt.	DT17	0.18	0.16	0.15	0.13	0.13
		(0.00)	(0.00)	(0.00)	(0.00)	(0.00)
Wet-lower-mid alt.	DT3	0.07	0.07	0.07	0.06	0.06
		(0.00)	(0.00)	(0.00)	(0.00)	(0.00)
Wet-lower-mid alt.	DT6	0.03	0.02	0.02	0.01	0.01
		(0.06)	(0.19)	(0.30)	(0.43)	(0.50)
Wet-lower-mid alt.	DT17	−0.28	−0.29	−0.30	−0.30	−0.30
		(0.00)	(0.00)	(0.00)	(0.00)	(0.00)
Low wetland	DT3	0.05	0.05	0.05	0.05	0.05
		(0.29)	(0.29)	(0.36)	(0.36)	(0.37)
Low wetland	DT6	0.03	0.03	0.02	0.02	0.02
		(0.55)	(0.62)	(0.63)	(0.68)	(0.68)
Low wetland	DT17	0.04	0.03	0.02	0.02	0.02
		(0.48)	(0.62)	(0.64)	(0.63)	(0.64)
Wet-upper-mid alt.	DT3	0.19	0.20	0.21	0.21	0.21
		(0.00)	(0.00)	(0.00)	(0.00)	(0.00)
Wet-upper-mid alt.	DT6	0.24	0.25	0.25	0.25	0.25
		(0.00)	(0.00)	(0.00)	(0.00)	(0.00)
Wet-upper-mid alt.	DT17	0.26	0.26	0.26	0.26	0.26
		(0.00)	(0.00)	(0.00)	(0.00)	(0.00)

More specifically, in dry mega-environments, the correlation between the rainfall index and yields range from −0.26 (*P* = 0.000) at the 10% quantile to −0.71 (*P* = 0.000) at the 50% quantile, and in wet environments, it ranges from 0.00 (*P* = 0.93) at the 10% quantile in the low wetland to −0.30 (*P* = 0.00) at the same quantile. Similarly, in the dry environments, correlation between the index and yield differential range from 0.13 (*P* = 0.000) to 0.68 (*P* = 0.000) and range from 0.02 (*P* = 0.63) to 0.26 (*P* = 0.00) in wet environments. Generally, the correlation between the yields and index is negative while that between the index and yield differential is mostly positive across mega-environments and quantile levels. Thus, revealing that an increase in the index value, which represents the proportion of cumulative rainfall shortfall over the growing season, leads to a decrease in the yields while an increase in the index value, overall, leads to an increase in the yield differential.

These results suggest a quadratic relationship between yield differentials and rainfall index as indicated in the stylised conceptual framework in Lybbert and Carter (2014) for bundling a drought tolerant maize variety and rainfall index insurance. In this relationship, net yields between a more drought tolerant variety and a less drought tolerant one approaches zero under (near) normal rainfall conditions. As rainfall decreases, drought tolerant variety yields become increasingly better than the base. Beyond a certain point, yields for the more drought tolerant variety also start to decrease and so thus the net yields. Under extremely severe drought conditions, yields from both varieties (and thus net yields) tend to zero.

Table 5 reports expected losses (in t/ha) by maize variety and insured risk quantile within each mega-environment while Table 6 reports corresponding actuarially fair premium rates. Results show significant heterogeneity in the expected loss and rate amongst the maize varieties both within and across mega-environments.

**TABLE 5:** Expected yield loss (t/ha)

Environment	Variety	10%	25%	35%	45%	50%
Dry lowland	DT3	0.03	0.23	0.36	0.47	0.54
Dry lowland	DT6	0.06	0.33	0.49	0.61	0.68
Dry lowland	DT11	0.04	0.25	0.39	0.5	0.57
Dry lowland	DT17	0.07	0.36	0.54	0.67	0.75
Dry-mid alt.	DT3	0.02	0.11	0.19	0.3	0.37
Dry-mid alt.	DT6	0.03	0.13	0.23	0.34	0.42
Dry-mid alt.	DT11	0.03	0.13	0.24	0.36	0.45
Dry-mid alt.	DT17	0.02	0.1	0.19	0.31	0.38
Wet-lower-mid alt.	DT3	0.1	0.24	0.34	0.45	0.51
Wet-lower-mid alt.	DT6	0.11	0.27	0.38	0.5	0.56
Wet-lower-mid alt.	DT11	0.12	0.28	0.41	0.54	0.61
Wet-lower-mid alt.	DT17	0.1	0.25	0.36	0.47	0.53
Low wetland	DT3	0.03	0.1	0.17	0.28	0.33
Low wetland	DT6	0.04	0.13	0.22	0.34	0.41
Low wetland	DT11	0.02	0.08	0.14	0.23	0.28
Low wetland	DT17	0.01	0.05	0.1	0.18	0.22
Wet-upper-mid alt.	DT3	0.05	0.2	0.29	0.41	0.48
Wet-upper-mid alt.	DT6	0.04	0.16	0.25	0.35	0.4
Wet-upper-mid alt.	DT11	0.04	0.17	0.25	0.35	0.41
Wet-upper-mid alt.	DT17	0.05	0.2	0.29	0.41	0.48

**TABLE 6:** Actuarially fair premium rates

Environment	Variety	10%	25%	35%	45%	50%
Dry lowland	DT3	0.2	0.18	0.19	0.19	0.19
Dry lowland	DT6	0.03	0.12	0.15	0.17	0.18
Dry lowland	DT11	NaN	0.72	0.38	0.28	0.26
Dry lowland	DT17	0.06	0.15	0.18	0.18	0.18
Dry-mid alt.	DT3	0.06	0.08	0.1	0.12	0.14
Dry-mid alt.	DT6	0.02	0.06	0.08	0.1	0.11
Dry-mid alt.	DT11	NaN	0.11	0.11	0.11	0.12
Dry-mid alt.	DT17	0.13	0.09	0.11	0.14	0.15
Wet-lower-mid alt.	DT3	0.08	0.11	0.13	0.14	0.15
Wet-lower-mid alt.	DT6	0.12	0.11	0.12	0.13	0.14
Wet-lower-mid alt.	DT11	0.15	0.15	0.16	0.16	0.17
Wet-lower-mid alt.	DT17	0.28	0.13	0.15	0.16	0.16
Low wetland	DT3	0.01	0.03	0.05	0.07	0.08
Low wetland	DT6	0.01	0.04	0.05	0.08	0.09
Low wetland	DT11	0.09	0.05	0.06	0.08	0.08
Low wetland	DT17	0.01	0.03	0.05	0.07	0.08
Wet-upper-mid alt.	DT3	0.04	0.08	0.1	0.11	0.12
Wet-upper-mid alt.	DT6	0.04	0.09	0.11	0.13	0.14
Wet-upper-mid alt.	DT11	0.07	0.1	0.11	0.13	0.14
Wet-upper-mid alt.	DT17	0.05	0.09	0.11	0.13	0.14

Overall, expected loss and premium rate decreases with decrease in insured risk layer (quantile). Results reported in both tables reveal distinct patterns in expected loss as we move from high to low risk quantiles; Table 6 shows relatively little or no change in fair premium rates in the mild to moderate (50%, 45% and 35%) risk quantile and a substantial decrease in rates from the moderate to severe risk quantiles. Comparing rates amongst varieties reveals DT6 has the lowest rates in dry lowlands and dry mid-altitudes while DT11 (and DT17) have the highest rates. Additionally, the magnitude of the differences in rates is remarkably higher in dry lowlands compared to wet mega-environments, and the differences in rates increases with decreases in the insured risk quantile. For example, the premium rate differential between DT6 and DT11 reveals that rates for DT6 are 44% to 500% lower in the dry lowland and only 9% to 83% lower in the dry mid-altitude, and except at the severe risk quantile, the rate differentials are minimal in the low wetland and wet-upper mid-altitude.

### 5.2 Potential welfare changes from bundling DTMV and index insurance

Table 7 presents certainty equivalent (CE) revenue and percentage change in CE revenue between DT11 and each maize variety. Akin to fair premium rates and yield (differentials), results show high variability in benefits of bundling DTMVs and RII amongst maize varieties within and across mega-environments. In dry environments, where it is most feasible to bundle DTMWs with RII, DT6 offers the highest CE revenue (1.6 to 3.54 currency units) and highest percentage increase in CE revenue (18.41% to 4,356.6%). In comparison, CE and percentage change in CE for DT6 are much lower (and negative in some risk layers) in low wetlands and wet-upper mid-altitude.

**TABLE 7:** Certainty equivalent (CE) revenue and percentage change in certainty equivalent (DCE) revenue

Environment	Variety	10%		25%		35%		45%		50%	
		CE	ΔCE	CE	ΔCE	CE	ΔCE	CE	ΔCE	CE	ΔCE
Dry lowland	DT3	0.55	NaN	0.61	24.88	1.49	1,975	2.05	335.5	2.31	121.43
Dry lowland	DT6	1.82	NaN	2.8	473.44	3.2	4,357	3.54	651.5	3.71	255.81
Dry lowland	DT17	1.56	NaN	2.18	347.15	2.81	3,820	3.33	607.6	3.59	244.53
Dry-mid alt.	DT3	0.18	NaN	−1.1	−160.34	1.53	−37.14	1.98	122.3	2.25	−85.05
Dry-mid alt.	DT6	1.60	NaN	2.49	36.63	2.89	18.41	3.25	263.7	3.43	−77.2
Dry-mid alt.	DT17	−0.54	NaN	0.76	−58.06	1.42	−42.03	2.02	125.8	2.28	−84.89
Wet-lower-mid alt.	DT3	11.2	1,942.5	1.86	113.06	2.31	27.84	2.77	15.38	2.94	13.95
Wet-lower-mid alt.	DT6	0.06	−88.91	−13.53	−1,651.1	1.41	−21.62	2.25	−6.12	2.78	7.46
Wet-lower-mid alt.	DT17	1.64	198.7	2.68	206.9	0.53	−70.42	2.18	−8.83	3.99	54.38
Low wetland	DT3	1.83	−307.4	2.86	285.24	3.34	105.6	3.78	66.69	3.99	56.11
Low wetland	DT6	2.76	−412.46	3.67	393.4	4.1	152.2	4.5	98.3	4.69	83.73
Low wetland	DT17	0.38	−143.35	1.49	100.79	2.01	23.54	2.46	8.58	2.68	5.06
Wet-upper-mid alt.	DT3	0.81	1,324.3	1.61	117.87	3.54	−130	2.78	35.64	2.99	38.36
Wet-upper-mid alt.	DT6	1.54	2,622.8	1.73	133.26	2.51	−121.3	2.29	11.59	2.48	14.56
Wet-upper-mid alt.	DT17	3.26	5,659.5	−0.26	−135.12	2.9	−124.6	0.86	−58.3	1.24	−42.69

Overall, DT6 offers the most benefits of bundling with a RII designed to insure the 50%, 45% and 35% drought risk quantiles in dry lowlands and dry mid-altitude.

Our results also show DT6 to be the most beneficial bundle in low wetlands based on similar drought risk layers while DT3 is the most beneficial in wet-lower mid-altitude. However, these results are less reliable compared to those obtained in dry lowlands and dry mid-altitude due to the weaker correlation between the index and yield (differential) in wet mega-environments.

In a nutshell, these results underscore the need to systematically bundle a specific variety and drought risk layer to insure in a given mega-environment in order to spur demand and facilitate scaling up and sustainability of crop insurance programmes. Failure to do so increases the chances of designing a contract that is far less beneficial and highly unattractive to farmers, thus leading to low demand for these policies.

## 6 Conclusion

Poor demand for index insurance with little potential for scalability and sustainability combined with higher vulnerability of rural households to drought has prompted research for improved risk management tools. We investigate the potential for bundling drought tolerant maize varieties with a simulated multi-site rainfall index insurance to better insure households against drought risk and facilitate scaling up and sustainability of farm risk management programmes. We use on-farm trial data conducted by CIMMYT and partners over 49 locations in Eastern and Southern Africa spanning 8 countries and 5 agro-mega environments with daily rainfall data to investigate the feasibility of such a bundle.

We find very high variation in the benefits of bundling a drought tolerant maize variety with a rainfall index insurance. The performance of the bundle depends on the maize tolerance to drought, the drought risk layer (trigger level) chosen, type of environment in which it is grown and the baseline maize variety to which it is compared. This implies that there are benefits to some combinations of DTMV and RII in a given environment based on the yield and premium cost at different trigger thresholds. The ultimate benefits of the bundle are realised by selecting an optimum trigger level conditional on the environment and the (baseline) maize variety.

We find a well-defined relationship between net yields and certainty equivalent estimates when comparing benefits of a drought tolerant maize and a baseline variety, making it feasible to select the best variety and an optimum insurance. As would be expected, the opportunities for defining an effcient bundle are greatest in dry mega-environments, and thus offer the best potential for scaling up programmes in these environments. In addition, the welfare benefits of bundling DTMV and RII are significantly higher in dry lowlands than low wetlands. Overall, we find high correlation between the index, yields and net yields at both high, medium and low trigger levels in dry lowlands, making basis risk less of a concern.

The policy implications of these results are significant, given that demand can be increased by systematically bundling the two technologies to produce contracts with lower premiums, and by increasing the area of coverage with a design that aims to insure all farmers within a dry lowland. In addition, our model framework can be used to recommend specific maize varieties that offer the most benefits in a given mega-environment with or without RII, thus facilitating decision-making under risk and multiple uncertainties to both farmers and policy-makers.

Additional research is needed to validate and convert the index developed in this study into a more dependable, practical product. For example, more accurate yield predictions could be obtained by using yield data for several years from the same farm fields, including other major input variables, such as those used in a crop growth model, as covariates in our model. Developing a model that allows for the joint simulation of space-time rainfall and other variables such as evapotranspiration will be very useful future research and will lay the groundwork for possible improvements to this study. Additional benefits of bundling DTMV with a multi-site index could also be obtained by comparing results derived using a multi-site rainfall index with those separately derived using a single index in each environment. In addition, selecting the insurance triggers in an optimisation process as opposed to taking a general approach (as done in this study) can produce more insightful results on the variation of the benefits and performance of the bundle across space. Finally, with the availability of suffcient data in the future, models that allow for site-specific effects of cumulative rainfall to be directly estimated are worth pursuing.

## Footnotes

2 For example, Drought Tolerant Maize for Africa (DTMA) Project funded by the Bill and Melinda Gates Foundation, the Howard G. Buffett Foundation, the US Agency for International Development, and the UK Department for International Development is jointly implemented by the International Maize and Wheat Improvement Center (CIMMYT) and the International Institute for Tropical Agriculture (IITA), in close collaboration with national agricultural research systems in 13 participating nations. Since 2001, at least 15 developing countries have introduced index insurance at the individual level and 20 at the institutional. Examples include AgroBrasil in Brazil; HARITA, Millenium Village and AXA Re in Ethiopia; BASIX, Pepsico, AIC and ICICI Lombard in India; Rockefeller and Kilimo Salama in Kenya; MicroEnsure, Opportunity International and World Bank in Malawi; Caribbean Catastrophe Risk Insurance Facility in 16 countries in the Caribbean; and the African Risk Capacity in Africa. See Carter *et al.* (2014) and Barnett *et al.* (2008) for details on the insurance programmes. The surge in interest in developing crop insurance markets in developing countries is driven by the need for more cost-effective *ex-post* disaster risk management strategy than post disaster recovery aid, and the lack of interest by commercial insurers for crop production risk due to systemic risk inherent in agriculture. This requires governments to play the role of an insurer and reinsurer at least in the initial phase of the programme. Index-based insurance is popular and highly preferred than farm-level contracts because it costs less to administer and is not susceptible to moral hazard or adverse selection.

3 We note that the insurance ratemaking process used in most pilot programmes allows for the premium estimates to be generated from a constrained optimization, and the final premium paid is reached through further negotiation between the insurer and the farmers (Osgood *et al.*, 2007).

4 R4 is a partnership between Oxfam America and the United Nations World Food Programme that integrates crop insurance, credits, savings and disaster risk reduction. ACRE Africa is a commercial company launched in 2014 that offers customised insurance products bundled with agricultural forecast, quality inputs and credit.

5 Data availability is the most fundamental problem encountered in pricing microinsurance contracts in developing countries (Biener, 2013), requiring a combination of innovative methods (e.g. Bayesian estimation, credibility theory and use of expert knowledge) for insurance rate-making.

6 An alternative, and theoretically preferable, model that captures different effects of predictors for the same varieties across sites is not feasible to simulate with our current data (see below).

7 Covariance functions that depend only on the distance metric to model spatial correlation between points are referred to as isotropic.

8 For more details about the step-by-step approach, see Wilks (1998) and Mhanna and Bauwens (2012). We updated and use R source codes from Mhanna and Bauwens (2012) available online to conduct our simulations.

9 Multi-phase weather indexes designed to capture the different crop growth stages and their water requirements are expected to better correlate with yields than a single-phase index.

10 Alternatively, rainfall shortfall and thus losses can be capped at a maximum by setting an exit rainfall amount. We also investigated a standardised index but it did not perform better.

11 An optimum trigger for each maize variety can be obtained by minimising the variance of yield losses (Osgood *et al.*, 2007). This can easily be obtained in this study by grid search since very few parameters are involved in the objective function.

12 Following past literature, we ignore the cost of administering the programmes. This assumption is also reasonable because the cost of administering an index-based insurance programme is minimal, as losses are not indemnified on individual farms. In practice, the cost can be priced into the gross premiums by adding it as a loading factor. Also, note that adding the cost of administering the programme will not change our main results.

## Supporting Information

**Table A1.** Model selection

**Table A2.** Posterior summary

**Table A3.** Summary of simulated yields (t/ha)

**Table A4.** Correlation between yields and rainfall index (*P*-value in parentheses)

**Figure S1.** Observed vs. Predicted yield at 8 locations within the wet lower mid-altitude environment.

## References

[cit0001] Barnett J. B., Barett C. B. and Skees J. R. ‘Poverty traps and index-based risk transfer products’, *World Development*, Vol. 36, (2008) pp. 1766–1785.

[cit0002] Biener C. ‘Pricing in microinsurance markets’, *World Development*, Vol. 41, (2013) pp. 132–144.

[cit0003] Binswanger-Mikhize P. H. ‘Is there too much hype about index-based agricultural insurance?’, *Journal of Development Studies*, Vol. 48, (2012) pp. 187–200.

[cit0004] Carriquiry M. A. and Osgood D. E. ‘Index insurance, probabilistic climate forecasts, and production’, *Journal of Risk & Insurance*, Vol. 79, (2012) pp. 287–300.

[cit0005] Carter M., de Janvry A., Sadoulet E. and Sarris A. *Index-based Weather Insurance for Developing Countries: A Review of Evidence and a Set of Propositions for Up Scaling*, Working Paper No. 111, 2014.

[cit0006] Clarke D. *A Theory of Rational Demand for Index Insurance*, Working Paper No. 572 (University of Oxford, Department of Economics, 2011).

[cit0007] Cole S., Giné X. and Vickery J. *How Does Risk Management Influence Production Decisions? Evidence from a field Experiment*, Working Paper No. 6546 (World Bank Policy Research, 2016).

[cit0008] Cooper P. J. M., Cappiello S. Vermeulen S. J., Campbell B. M., Zougmoré R. and Kinyangi *Large-scale Implementation of Adaptation and Mitigation Actions in Agriculture*, Working Paper No. 50 (CGIAR Research Program on Climate Change, Agriculture and Food Security, 2013).

[cit0009] de Janvry A., Dequiedt V. and Sadoulet E. ‘The demand for insurance against common shocks’, *Journal of Development Economics*, Vol. 106, (2014) pp. 227–238.

[cit0010] Dercon S., Hill R. V., Clarke D., Outes-Leon I. and Taffesse A. S. ‘Offering rainfall insurance to informal insurance groups: Evidence from a field experiment in Ethiopia’, *Journal of Development Economics*, Vol. 106, (2014) pp. 132–143.

[cit0011] Diiro G. M. *Impact of Off-farm Income on Technology Adoption Intensity and Productivity: Evidence from Rural Maize Farmers in Uganda*, Working Paper No. 11 (Uganda strategy support program, IFPRI: 1 2013).

[cit0012] Eswaran M. and Kotwal A. ‘Implications of credit market constraints for risk behavior in less developed economies’, *Oxford Economic Papers*, Vol. 42, (1990) pp. 473–482.

[cit0013] FAO. *Rural Household Vulnerability and Insurance against Commodity Risks: Evidence from the United Republic of Tanzania*. FAO Commodities and Trade Technical Paper No. 10, 2007 Available at: http://www.fao.org/3/a1310e/a1310e00.htm (last accessed: 8 May 2019).

[cit0014] FAO, WFP and IFAD *The State of Food Insecurity in the World 2012. Economic Growth is Necessary but not Suffcient to Accelerate Reduction of Hunger and Malnutrition* (FAO, Rome, 2012). Available at: http://www.fao.org/publications/sofi/en/ (last accessed: 8 May 2019). 10.3945/an.112.003343 PMC3648734 23319131

[cit0015] Finley A. O., Banerjee S. and Carlin B. ‘spbayes: R package for univariate and multivariate hierarchical point-referenced spatial models’, *Journal of Statistical Software*, Vol. 19, (2007) pp. 1–24. 21494410 10.18637/jss.v019.i04 PMC3074178

[cit0016] Finley A. O., Banerjee S. and McRoberts R. ‘A Bayesian approach to quantifying uncertainty in multi-source forest area estimates’, *Environmental and Ecological Statistics*, Vol. 15, (2008) pp. 241–258.

[cit0017] Fisher M., Abate T., Lunduka R. W., Asnake W., Alemayehu Y. and Madulu R. B. ‘Drought tolerant maize for farmer adaptation to drought in sub-Saharan Africa: Determinants of adoption in eastern and southern Africa’, *Climatic Change*, Vol. 133, (2015) pp. 283–299.

[cit0018] Gelfand A. E. and Ghosh S. K. ‘Model choice: A minimum posterior predictive loss approach’, *Biometrika*, Vol. 85, (1998) pp. 1–11.

[cit0019] Gelman A., Carlin J. B., Stern H. S. and Rubin D. B. *Bayesian Data Analysis*, 2nd edn (Boca Raton, FL: Chapman & Hall/CRC, 2003).

[cit0020] Gneiting T. and Raftery A. E. ‘Strictly proper scoring rules, prediction, and estimation’, *Journal of the American Statistical Association*, Vol. 102, (2007) pp. 359–378.

[cit0021] Hess U. and Syroka J. *Weather Index-based Insurance in Southern Africa. The Case of Malawi*, Discussion Paper No. 13 (ARD, The World Bank, Washington, DC, 2006).

[cit0022] IRI *Designing Index-based Weather Insurance for Farmers in Adiha, Ethiopia*. IRI Technical Report No. 09-04, 2009 Available at: https://academiccommons.columbia.edu/doi/10.7916/D8G166RB/download (last accessed: 8 May 2019).

[cit0023] Jacoby H. and Skoufias E. ‘Risk, financial markets and human capital in a developing country’, *Review of Economic Studies*, Vol. 64, (1997) pp. 311–345.

[cit0024] Langyintuo A. S., Mwangi W., Diallo A. O., MacRobert J., Dixon J. and Banziger M. ‘Challenges of the maize seed industry in eastern and southern Africa: A compelling case for private-public intervention to promote growth’, *Food Policy*, Vol. 35, (2010) pp. 323–331.

[cit0025] Lybbert T. J. and Carter M. R. ‘Bundling drought tolerance and index insurance to reduce household vulnerability to drought’, *Sustainable Economic Development: Resources, Environment, and Insitutions*, Elsevier Inc., (2014) pp. 401–414 10.1016/B978-0-12-800347-3.00022-4

[cit0026] Mhanna M. and Bauwens, W. ‘A stochastic space-time model for the generation of daily rainfall in the Gaza strip’, *International Journal of Climatology*, Vol. 32, (2012) pp. 1098–1112.

[cit0027] Molini, V. M., Keyzer M., Van der Boom G. and Zant W. ‘Creating safety nets through semi-parametric index-based insurance: A simulation for northern Ghana’, *Agricultural Finance Review*, Vol. Spring, (2008) pp. 223–225.

[cit0028] Osgood D. E., McLaurin M., Carriquiry M., Mishra A., Fiondella F., Hansen J., Peterson N. and Ward N. (2007). *Designing Weather Insurance Contracts for Farmers in Malawi, Tanzania, and Kenya*. IRI Technical Report No. 07-02. Available at: https://iri.columbia.edu/~deo/IRI-CRMG-Africa-Insurance-Report-6-2007/IRI-CRMG-Kenya-Tanzania-Malawi-Insurance-Report-6-2007.pdf (last accessed: 8 May 2019).

[cit0029] Rosenzweig M. and Wolpin, K. ‘Credit market constraints, consumption smoothing and the accumulation of durable production assets in low-income countries: Investment in bullocks in India’, *Journal of Political Economy*, Vol. 101, (1993) pp. 223–244.

[cit0030] Skees J. R. and Barnett J. B. ‘Enhancing micro-finance using index-based risk transfer products’, *Agricultural Finance Review*, Vol. 66, (2006) pp. 235–250.

[cit0031] Skees J., Hartell J. and Murphy A. ‘Using index-based risk transfer products to facilitate micro lending in Peru and Vietnam’, *American Journal of Agricultural Economics*, Vol. 89, (2007) pp. 1255–1261.

[cit0032] Traerup S. L. ‘Informal networks and resilience to climate change impacts: A collective approach to index insurance’, *Global Environmental Change*, Vol. 22, (2011) pp. 255–267.

[cit0033] Wackernagel H. *Multivariate Geostatistics; An Introduction with Applications*, 3rd edn (New York, US: Springer, 2003).

[cit0034] Wilks D. S. ‘Multisite generalization of a daily stochastic precipitation generation model’, *Journal of Hydrology*, Vol. 210, (1998) pp. 178–191.

