# Supplementary material for: Multi-Site Bundling of Drought Tolerant Maize Varieties and Index Insurance
Source: J Agric Econ. 2019 Jun 4;71(1):1–21. doi: 10.1111/1477-9552.12344 (PMC7198119; doi:10.1111/1477-9552.12344)
Supplement: Supplementary file 1 [file JAE-2019-1477-9552-12344-s1.docx]

Multi-Site Bundling of Drought Tolerant Maize Varieties and Index Insurance

Sebastain N. Awondo, Genti Kostandini, Peter Setimela and Olaf Erentein

**Online Appendix**

Data for this study comes from on-farm trials conducted by CIMMYT and partners in 2011 in 49 farmers’ ﬁelds under farmer management across 8 countries in Eastern and Southeastern African countries; 20 of the trials were conducted within 5 communities (Bikita, Zaka, Gokwe, Mtoko and Mrewa) in Zimbabwe; 8 within 8 communities (UlongaEPA, RiviriviEPA, Golomoti, GolomotiEPA, Chipoka, ChipokaEPASal., PhalulaBalaka and ChipokaEPASal2.) in Malawi; 4 trials within 4 communities in Zambia (Chisamba, LusakaWest, MonzeEast and Monze), Uganda (Iganga, Gulu, Masindi and Wakiso), and Ethiopia (Ethiopia1, Ethiopia2, Ethiopia3 and Bofa); 3 trials within 3 communities (Matsinnho-Gond., VanduziManica and Gondola-Caf.) in Mozambique; 5 trials within 5 communities (Kipini, Malava, Bungoma, Alupe and KibosPrison) in Kenya, and 1 trial in Tanzania (Bomangombe). The 49 farm ﬁelds were associated with 5 different agro-mega environments: dry lowland, dry-mid altitude, wet-lower-mid altitude, low wetland and wet-upper-mid altitude. Nineteen different improved maize varieties including hybrids, open-pollinated varieties, commercial varieties, and local varieties (simply referred to here as DT1 to DT19) plus one local maize variety were tested on a farm plot at each location. Each farm represented a block in a randomised complete-block design.

During the year that the trials were conducted only 13 locations actually experienced water stress levels in 2011, making it difficult to properly evaluate genotype performance and stability under various drought severity levels. In addition, the local variety, which varies by location, makes it difficult to model in a multivariate framework with the other 19 varieties. The results presented below are based on 19 varieties excluding the local variety.

Table A1: Model selection

| **X** | pD | DIC | G | D |
| --- | --- | --- | --- | --- |
| Exponential |  |  |  |  |
| **X_1_** | 525.98 | 1,270.20 | 243.23 | 1,714.66 |
| **X_2_** | 473.79 | 1,227.45 | 275.39 | 1,744.57 |
| **X_3_** | 495.28 | 1,226.13 | 256.50 | 1,714.45 |
| **X_4_** | 624.07 | 1,307.57 | 197.31 | 1,817.35 |
| **X_5_** | 510.62 | 1,268.30 | 254.34 | 1,731.36 |
| **X_6_** | 621.32 | 1,365.01 | 210.30 | 1,739.22 |
| **X_7_** | 644.01 | 1,416.29 | 197.20 | 1,835.58 |
| Spherical |  |  |  |  |
| **X_1_** | 522.12 | 1,318.28 | 247.48 | 1,810.37 |
| **X_2_** | 508.84 | 1,267.19 | 261.36 | 1,728.54 |
| **X_3_** | 487.39 | 1,236.36 | 263.73 | 1,737.32 |
| **X_4_** | 633.08 | 1,301.63 | 252.85 | 1,740.64 |
| **X_5_** | 545.49 | 1,271.19 | 208.58 | 1,687.14 |
| **X_6_** | 648.76 | 1,301.86 | 203.74 | 1,577.56 |
| **X_7_** | 620.00 | 1,563.47 | 296.81 | 2,221.48 |
| Gaussian |  |  |  |  |
| **X_1_** | 524.19 | 1,282.74 | 238.34 | 1,742.25 |
| **X_2_** | 464.54 | 1,204.69 | 272.74 | 1,725.44 |
| **X_3_** | 463.76 | 1,189.51 | 283.23 | 1,691.41 |
| **X_4_** | 623.46 | 1,200.20 | 219.57 | 1,628.59 |
| **X_5_** | 522.76 | 1,275.28 | 233.50 | 1,740.30 |
| **X_6_** | 615.93 | 1,412.92 | 236.06 | 1,811.44 |
| **X_7_** | 647.75 | 1,398.02 | 223.58 | 1,794.10 |
| Mat´ern |  |  |  |  |
| **X_1_** | 544.65 | 1,330.34 | 236.20 | 1,777.53 |
| **X_2_** | 491.51 | 1,290.50 | 282.68 | 1,824.77 |
| **X_3_** | 500.18 | 1,270.75 | 230.72 | 1,798.57 |
| **X_4_** | 594.37 | 1,326.88 | 251.27 | 1,808.69 |
| **X_5_** | 519.42 | 1,266.76 | 243.30 | 1,714.55 |
| **X_6_** | 638.53 | 1,220.43 | 185.60 | 1,494.28 |

**X_7_** 623.80 1,436.12 291.08 1,836.62

DIC=Deviance information criterion; pD=Effective number of parameters; G=Sum of squared error and D=Deviance criterion.


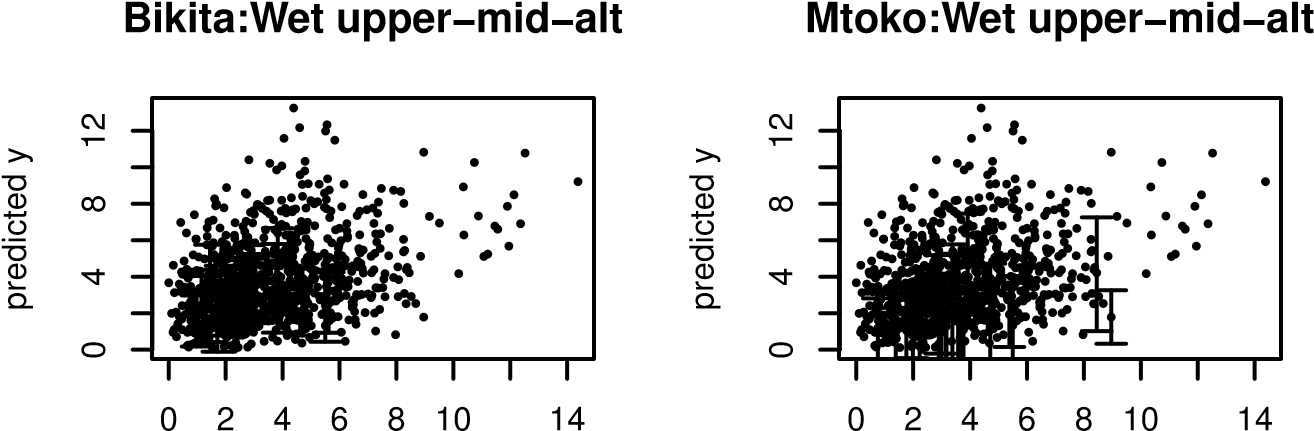


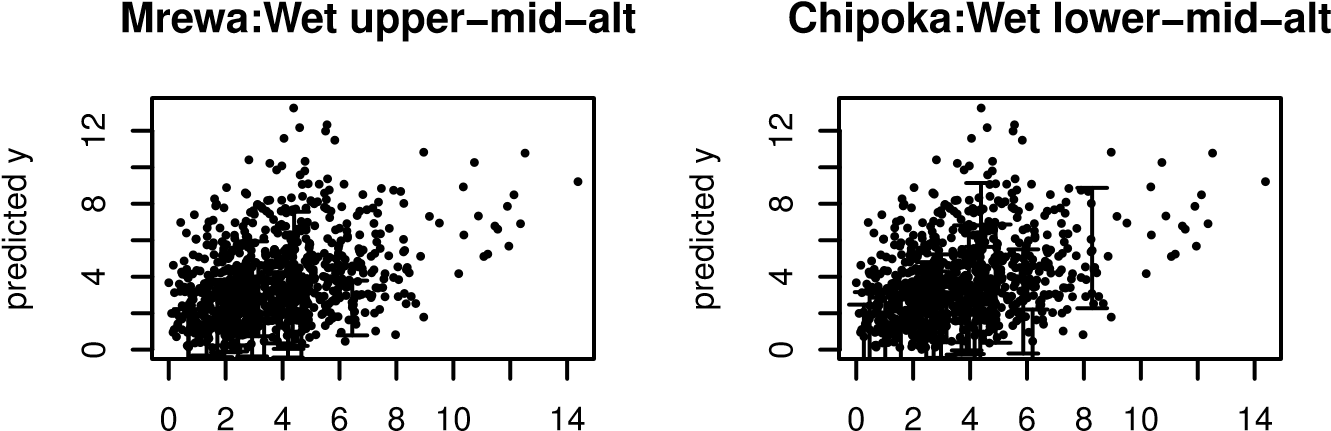
 Observed Y Observed Y

Observed Y Observed Y


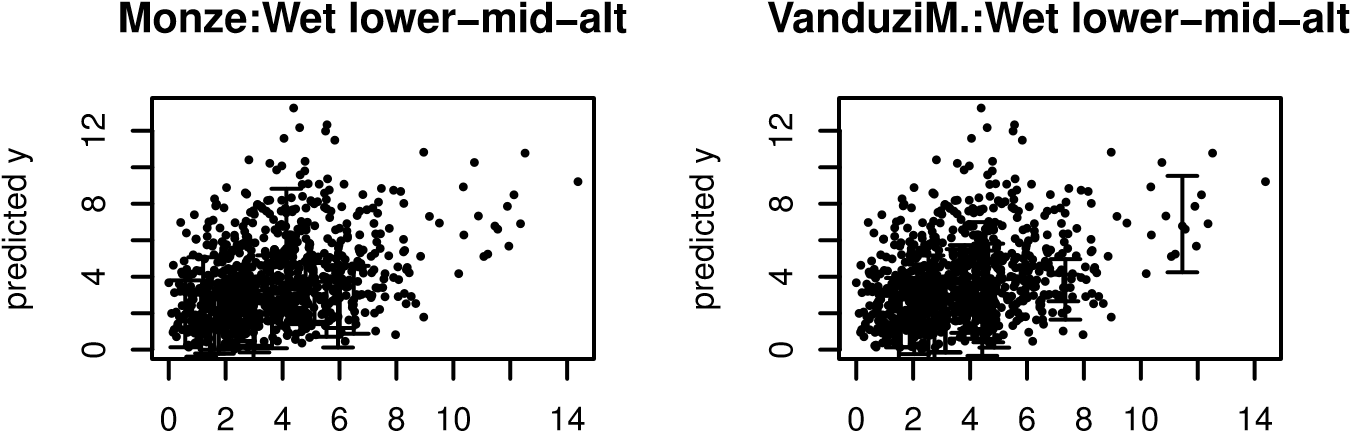


Observed Y Observed Y

**Figure S1:** Observed Vs Predicted yield at 8 locations within the wet lower mid-altitude environment.

Table A2: Posterior summary

| Model |  | *β*_0_ |  |  |  | *βP RCP* |  |  |  | *µ* |  |  |  | *θ* |  |  |  | Ψ |  |
| --- | --- | --- | --- | --- | --- | --- | --- | --- | --- | --- | --- | --- | --- | --- | --- | --- | --- | --- | --- |
|  | 50% | 2.5% | 97.5% |  | 50% | 2.5% | 97.5% |  | 50% | 2.5% | 97.5% |  | 50% | 2.5% | 97.5% |  | 50% | 2.5% | 97.5% |
|  |  |  |  |  |  |  |  |  |  |  |  |  |  |  |  |  |  |  |  |
| mod1 | 2.474 | 0.334 | 4.469 |  | 0.002 | -0.000 | 0.005 |  | 0.01 | 0.01 | 0.02 |  | 12.00 | 12.00 | 12.00 |  | 0.95 | 0.94 | 1.06 |
| mod2 | 2.335 | 0.173 | 4.559 |  | 0.002 | -0.001 | 0.005 |  | 1.88 | 1.80 | 1.98 |  | 4.60 | 4.60 | 8.00 |  | 0.42 | 0.38 | 0.42 |
| mod3 | 1.391 | -0.865 | 3.804 |  | 0.005 | 0.002 | 0.008 |  | 0.02 | 0.00 | 0.03 |  | 5.06 | 5.06 | 8.71 |  | 0.30 | 0.30 | 0.41 |
| mod4 | 2.737 | 0.255 | 5.231 |  | 0.002 | -0.001 | 0.006 |  | 0.67 | 0.05 | 0.67 |  | 5.82 | 4.40 | 5.82 |  | 0.97 | 0.97 | 1.13 |
| mod5 | 1.731 | -0.541 | 3.841 |  | 0.003 | 0.001 | 0.006 |  | 0.01 | 0.00 | 0.01 |  | 11.08 | 10.33 | 11.19 |  | 1.17 | 1.12 | 1.20 |
| mod6 | 1.555 | -0.724 | 3.722 |  | 0.003 | 0.000 | 0.007 |  | 1.99 | 1.93 | 1.99 |  | 8.46 | 4.87 | 8.46 |  | 0.77 | 0.77 | 0.82 |
| mod7 | 2.974 | 0.722 | 5.161 |  | 0.005 | 0.002 | 0.008 |  | 0.52 | 0.05 | 0.52 |  | 10.77 | 8.91 | 10.97 |  | 1.12 | 1.08 | 1.22 |
| mod8 | 2.022 | -0.616 | 4.411 |  | 0.003 | -0.000 | 0.007 |  | 1.39 | 1.39 | 1.89 |  | 11.96 | 11.96 | 11.98 |  | 2.13 | 2.13 | 2.28 |
| mod9 | 1.202 | -1.636 | 4.077 |  | 0.004 | 0.000 | 0.008 |  | 0.76 | 0.76 | 1.59 |  | 5.60 | 4.10 | 5.60 |  | 1.80 | 1.80 | 2.26 |
| mod10 | 2.408 | -0.202 | 5.181 |  | 0.004 | 0.000 | 0.008 |  | 2.00 | 2.00 | 2.00 |  | 4.65 | 4.49 | 4.81 |  | 1.18 | 1.15 | 1.18 |
| mod11 | 3.227 | 0.625 | 5.802 |  | 0.006 | 0.002 | 0.009 |  | 0.00 | 0.00 | 0.06 |  | 5.04 | 4.12 | 5.04 |  | 0.60 | 0.55 | 0.64 |
| mod12 | 2.283 | 0.037 | 4.524 |  | 0.002 | -0.001 | 0.005 |  | 0.17 | 0.17 | 0.27 |  | 10.72 | 9.76 | 11.08 |  | 1.06 | 1.06 | 1.10 |
| mod13 | 1.983 | 0.039 | 3.893 |  | 0.004 | 0.002 | 0.007 |  | 0.10 | 0.05 | 0.11 |  | 8.78 | 8.78 | 9.17 |  | 0.69 | 0.69 | 0.76 |
| mod14 | 2.386 | 0.188 | 4.562 |  | 0.002 | -0.001 | 0.005 |  | 0.80 | 0.72 | 0.98 |  | 4.49 | 4.49 | 6.34 |  | 0.25 | 0.24 | 0.26 |
| mod15 | 1.653 | -0.180 | 3.483 |  | 0.003 | 0.000 | 0.005 |  | 0.00 | 0.00 | 0.01 |  | 9.05 | 9.05 | 10.74 |  | 0.68 | 0.66 | 0.68 |
| mod16 | 1.180 | -1.621 | 4.091 |  | 0.003 | -0.001 | 0.007 |  | 0.94 | 0.94 | 1.27 |  | 8.44 | 8.26 | 10.08 |  | 0.39 | 0.39 | 0.42 |
| mod17 | 2.578 | -0.320 | 5.459 |  | 0.004 | 0.000 | 0.008 |  | 0.00 | 0.00 | 0.00 |  | 4.52 | 4.30 | 4.68 |  | 1.24 | 1.05 | 1.24 |
| mod18 | 1.583 | -1.215 | 4.623 |  | 0.004 | 0.000 | 0.008 |  | 1.60 | 1.40 | 1.94 |  | 10.75 | 10.75 | 11.62 |  | 0.75 | 0.71 | 0.92 |
| mod19 | 1.951 | -0.639 | 4.474 |  | 0.006 | 0.002 | 0.009 |  | 0.04 | 0.04 | 0.23 |  | 4.56 | 4.20 | 4.56 |  | 0.53 | 0.53 | 0.55 |

*β*_0_=intercept estimate; *β_PRCP_ =* cumulative rainfall estimate; *µ* = smoothness parameter for Mat´ern correlation function; Φ=correlation decay parameter; Ψ = dispersion parameter.

Table A3: Summary of simulated yields (t/ha)

| Environment | Variety | 10% | 25% | 35% | 45% | 50% | 97.50% |
| --- | --- | --- | --- | --- | --- | --- | --- |
|  |  |  |  |  |  |  |  |
| Dry lowland | DT3 | 0.17 | 1.28 | 1.88 | 2.49 | 2.82 | 7.58 |
| Dry lowland | DT6 | 1.93 | 2.84 | 3.29 | 3.7 | 3.91 | 6.66 |
| Dry lowland | DT11 | 0 | 0.35 | 1.04 | 1.79 | 2.2 | 10.65 |
| Dry lowland | DT17 | 1.15 | 2.37 | 3.05 | 3.77 | 4.17 | 8.85 |
| Dry-mid alt. | DT3 | 0.38 | 1.37 | 1.88 | 2.38 | 2.64 | 9.8 |
| Dry-mid alt. | DT6 | 1.49 | 2.37 | 2.9 | 3.5 | 3.85 | 7.93 |
| Dry-mid alt. | DT11 | 0 | 1.21 | 2.2 | 3.19 | 3.71 | 11.39 |
| Dry-mid alt. | DT17 | 0.16 | 1.17 | 1.7 | 2.21 | 2.49 | 11.34 |
| Wet-lower-mid alt. | DT3 | 1.28 | 2.19 | 2.71 | 3.23 | 3.49 | 7.08 |
| Wet-lower-mid alt. | DT6 | 0.98 | 2.54 | 3.23 | 3.74 | 3.97 | 9.65 |
| Wet-lower-mid alt. | DT11 | 0.79 | 1.9 | 2.59 | 3.3 | 3.68 | 11.64 |
| Wet-lower-mid alt. | DT17 | 0.37 | 1.86 | 2.44 | 2.99 | 3.28 | 9.96 |
| Low wetland | DT3 | 1.88 | 2.9 | 3.39 | 3.82 | 4.03 | 7.34 |
| Low wetland | DT6 | 2.63 | 3.56 | 4.04 | 4.5 | 4.73 | 8.91 |
| Low wetland | DT11 | 0.21 | 1.54 | 2.26 | 3.03 | 3.41 | 7.91 |
| Low wetland | DT17 | 0.47 | 1.54 | 2.04 | 2.49 | 2.71 | 6.16 |
| Wet-upper-mid alt. | DT3 | 1.18 | 2.45 | 3.07 | 3.61 | 3.85 | 7.33 |
| Wet-upper-mid alt. | DT6 | 1 | 1.75 | 2.18 | 2.63 | 2.86 | 7.22 |
| Wet-upper-mid alt. | DT11 | 0.64 | 1.7 | 2.24 | 2.73 | 2.97 | 7.65 |
| Wet-upper-mid alt. | DT17 | 0.94 | 2.19 | 2.65 | 3.1 | 3.33 | 9.26 |

Table A4: Correlation between yields and rainfall index (*p*-values in parentheses)

| Environment | Variety | 10% | 25% | 35% | 45% | 50% |
| --- | --- | --- | --- | --- | --- | --- |
|  |  |  |  |  |  |  |
| Dry lowland | DT3 | -0.46  (0.00) | -0.48  (0.00) | -0.49  (0.00) | -0.50  (0.00) | -0.50  (0.00) |
| Dry lowland | DT6 | -0.26  (0.00) | -0.31  (0.00) | -0.34  (0.00) | -0.36  (0.00) | -0.37  (0.00) |
| Dry lowland | DT11 | -0.45  (0.00) | -0.45  (0.00) | -0.45  (0.00) | -0.45  (0.00) | -0.45  (0.00) |
| Dry lowland | DT17 | -0.61  (0.00) | -0.60  (0.00) | -0.60  (0.00) | -0.59  (0.00) | -0.59  (0.00) |
| Dry-mid alt. | DT3 | -0.73  (0.00) | -0.73  (0.00) | -0.73  (0.00) | -0.73  (0.00) | -0.73  (0.00) |
| Dry-mid alt. | DT6 | -0.67  (0.00) | -0.67  (0.00) | -0.67  (0.00) | -0.67  (0.00) | -0.67  (0.00) |
| Dry-mid alt. | DT11 | -0.71  (0.00) | -0.71  (0.00) | -0.71  (0.00) | -0.71  (0.00) | -0.71  (0.00) |
| Dry-mid alt. | DT17 | -0.73  (0.00) | -0.73  (0.00) | -0.73  (0.00) | -0.73  (0.00) | -0.73  (0.00) |
| Wet-lower-mid alt. | DT3 | 0.02  (0.24) | 0.02  (0.17) | 0.02  (0.16) | 0.02  (0.15) | 0.02  (0.15) |
| Wet-lower-mid alt. | DT6 | -0.03  (0.05) | -0.04  (0.02) | -0.04  (0.01) | -0.04  (0.01) | -0.05  (0.01) |
| Wet-lower-mid alt. | DT11 | -0.04  (0.01) | -0.04  (0.01) | -0.04  (0.01) | -0.04  (0.02) | -0.04  (0.02) |
| Wet-lower-mid alt. | DT17 | -0.26  (0.00) | -0.27  (0.00) | -0.27  (0.00) | -0.27  (0.00) | -0.27  (0.00) |
| Low wetland | DT3 | 0.00  (0.93) | 0.02  (0.77) | 0.00  (0.92) | 0.01  (0.86) | 0.01  (0.91) |
| Low wetland | DT6 | -0.01  (0.90) | -0.01  (0.84) | -0.00  (0.93) | -0.01  (0.85) | -0.01  (0.84) |
| Low wetland | DT11 | -0.04  (0.39) | -0.04  (0.41) | -0.04  (0.44) | -0.04  (0.45) | -0.04  (0.45) |
| Low wetland | DT17 | -0.03  (0.58) | -0.08  (0.11) | -0.08  (0.13) | -0.07  (0.19) | -0.07  (0.16) |
| Wet-upper-mid alt. | DT3 | -0.15  (0.00) | -0.15  (0.00) | -0.16  (0.00) | -0.16  (0.00) | -0.16  (0.00) |
| Wet-upper-mid alt. | DT6 | -0.07  (0.00) | -0.08  (0.00) | -0.08  (0.00) | -0.09  (0.00) | -0.09  (0.00) |
| Wet-upper-mid alt. | DT11 | -0.28  (0.00) | -0.29  (0.00) | -0.30  (0.00) | -0.30  (0.00) | -0.30  (0.00) |
| Wet-upper-mid alt. | DT17 | -0.05  (0.00) | -0.06  (0.00) | -0.06  (0.00) | -0.06  (0.00) | -0.06  (0.00) |
